# A novel intercostal approach for single-port robotic right upper lobectomy: Surgical technique

**DOI:** 10.1016/j.xjtc.2025.102168

**Published:** 2025-12-02

**Authors:** Ramaswamy Rajendran, Ernest Cun Wang Ong, Yin-Kai Chao

**Affiliations:** a Department of Thoracic Surgery, Santosham Chest Hospital, Chennai, India; b Department of Thoracic Surgery, Chang Gung Memorial Hospital-Linkou, Taoyuan, Taiwan; c Department of Upper Gastrointestinal Surgery, National Cancer Institute, Ministry of Health Malaysia, Putrajaya, Malaysia

**Figure:**
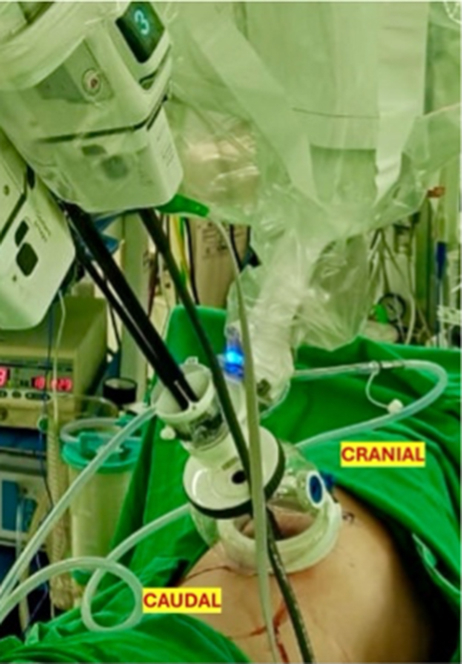
Single-port robotic right upper lobectomy via an intercostal access.

**Central Message**

Technique of performing an intercostal uniportal robotic right upper lobectomy using the da Vinci SP system (Intuitive Surgical).

The da Vinci SP system (Intuitive Surgical) represents a major advancement in minimally invasive surgery that allows complex procedures through a single incision. Since its introduction for lung resection, all published cases have employed the subcostal approach.^1^^,^^2^ This consistent choice reflects anatomical constraints because the SP cannula diameter typically exceeds the width of the intercostal space, making subcostal insertion technically more feasible.^2^

The subcostal entry point is relatively distant from the pulmonary hilum, which can complicate complex hilar dissection and impede prompt proximal vascular control in the event of unexpected bleeding. Recognizing these limitations, an intercostal access may provide direct anatomical alignment with the hilum and improved surgical control. We present a case of uniportal robotic right upper lobectomy performed entirely through an intercostal incision, demonstrating the technical feasibility of this approach.

## Case Report

A 58-year-old woman with cT1bN0 adenocarcinoma underwent uniportal robotic right upper lobectomy with systematic mediastinal lymphadenectomy using the da Vinci SP system via an intercostal approach. Informed consent was obtained for publication of clinical data. As per institutional policy, ethics approval was not required for this single-patient case report.

**Figure 1:**
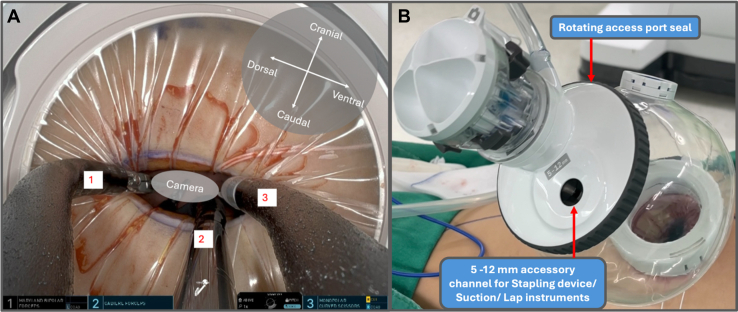
A, Float docking technique. B, da Vinci SP Access Port Kit (Intuitive Surgical).

**Figure 2:**
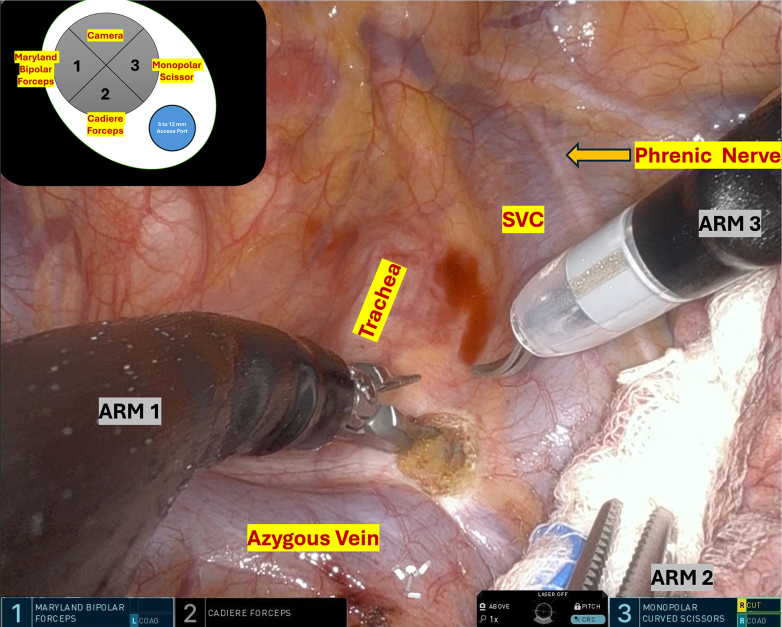
Right paratracheal lymph node dissection. *SVC*, Superior vena cava.

After intubation with a double-lumen endotracheal tube, the patient was positioned in the left lateral decubitus position. A 4-cm incision was created at the seventh intercostal space along the posterior axillary line. The da Vinci SP Access Port Kit was inserted and connected to an insufflator, with the single 2.5-cm trocar docked to the da Vinci SP side cart arm. Using the float docking technique, the 3 instrument arms were fully deployed before entry and then positioned at the extreme right, left, and inferior margins of the intercostal site (Figure 1, *A*, and Video 1). This configuration provided sufficient clearance for insertion of the 1.6-cm camera while minimizing instrument crowding at the chest wall. The surgical technique is demonstrated in Video 2. Three robotic instruments—a Cadiere forceps, monopolar curved scissors, and Maryland bipolar forceps—were utilized. The surgical sequence followed standard multiport robotic principles. After completing mediastinal lymph node dissection (Figure 2), meticulous hilar dissection was performed to create adequate space for safe stapler introduction. The handheld powered endovascular stapling device was then inserted through the rotating accessory channel of the SP access port kit (Figure 1, *B*). Total operative time was 187 minutes, with docking and console times of 2 and 172 minutes, respectively. The chest tube was removed on postoperative day 1, and the patient was discharged on postoperative day 3 without complications. Histopathological examination revealed poorly differentiated adenocarcinoma pT1bN0 (28 lymph nodes examined) with negative resections margins.

## Discussion

Compared with the subcostal route, the intercostal approach offers a shorter working distance with favorable angles to the hilum, enabling improved visualization and efficient management of potential vascular emergencies. However, the narrow vertical dimension of the intercostal space presents an obvious technical challenge for a 2.5-cm cannula. In this scenario, successful intercostal uniportal access requires the instrument assembly to be distributed horizontally to compensate for the constrained vertical height of the intercostal space. To accomplish this, the float docking technique is employed. Float docking entails predeployment of all 3 instrument arms and the camera outside the chest, minimizing intrathoracic volume required for docking, reduces the friction against the chest wall, and torque at the access port.

The intercostal uniportal approach has notable limitations. First, this approach requires a minimum 4-cm incision to allow safe and stable SP instrument articulation, which currently limits further reduction in incision length. Second, although a dedicated SP stapler has recently been approved in the United States, it is not yet commercially available in our region. Accordingly, our technique was optimized to utilize the rotating accessory channel of the SP access port kit for the introduction of conventional staplers without creating an additional incision. We anticipate that once the dedicated SP stapler becomes available locally, console-controlled stapling may simplify the workflow, reduce bedside assistant involvement, and further enhance procedural reproducibility. In addition, we acknowledge that compared with the subcostal approach, the intercostal trajectory may increase the risk of intercostal nerve irritation, which in some patients may contribute to a higher likelihood of long-term neuralgia. This consideration should be taken into account when selecting the optimal access route for individual patients. Further evaluation with longer-term sensory follow-up will be necessary to better define the clinical influence of intercostal access on postoperative neural outcomes.

## Conclusions

Intercostal uniportal robotic approach using the da Vinci SP system is safe and technically feasible for anatomical lung resection.

## Conflict of Interest Statement

The authors reported no conflicts of interest.

The *Journal* policy requires editors and reviewers to disclose conflicts of interest and to decline handling or reviewing manuscripts for which they may have a conflict of interest. The editors and reviewers of this article have no conflicts of interest.

## Supplementary Data

Video 1 Float docking technique. Video available at: https://www.jtcvs.org/article/S2666-2507(25)00549-8/fulltext.

Video 2 Intercostal approach for single port robotic right upper lobectomy. Video available at: https://www.jtcvs.org/article/S2666-2507(25)00549-8/fulltext.
