# Supplementary figures and images for: A novel intercostal approach for single-port robotic right upper lobectomy: Surgical technique
Source: JTCVS Tech. 2025 Dec 2;35:102168. doi: 10.1016/j.xjtc.2025.102168 (PMC12881822; doi:10.1016/j.xjtc.2025.102168)

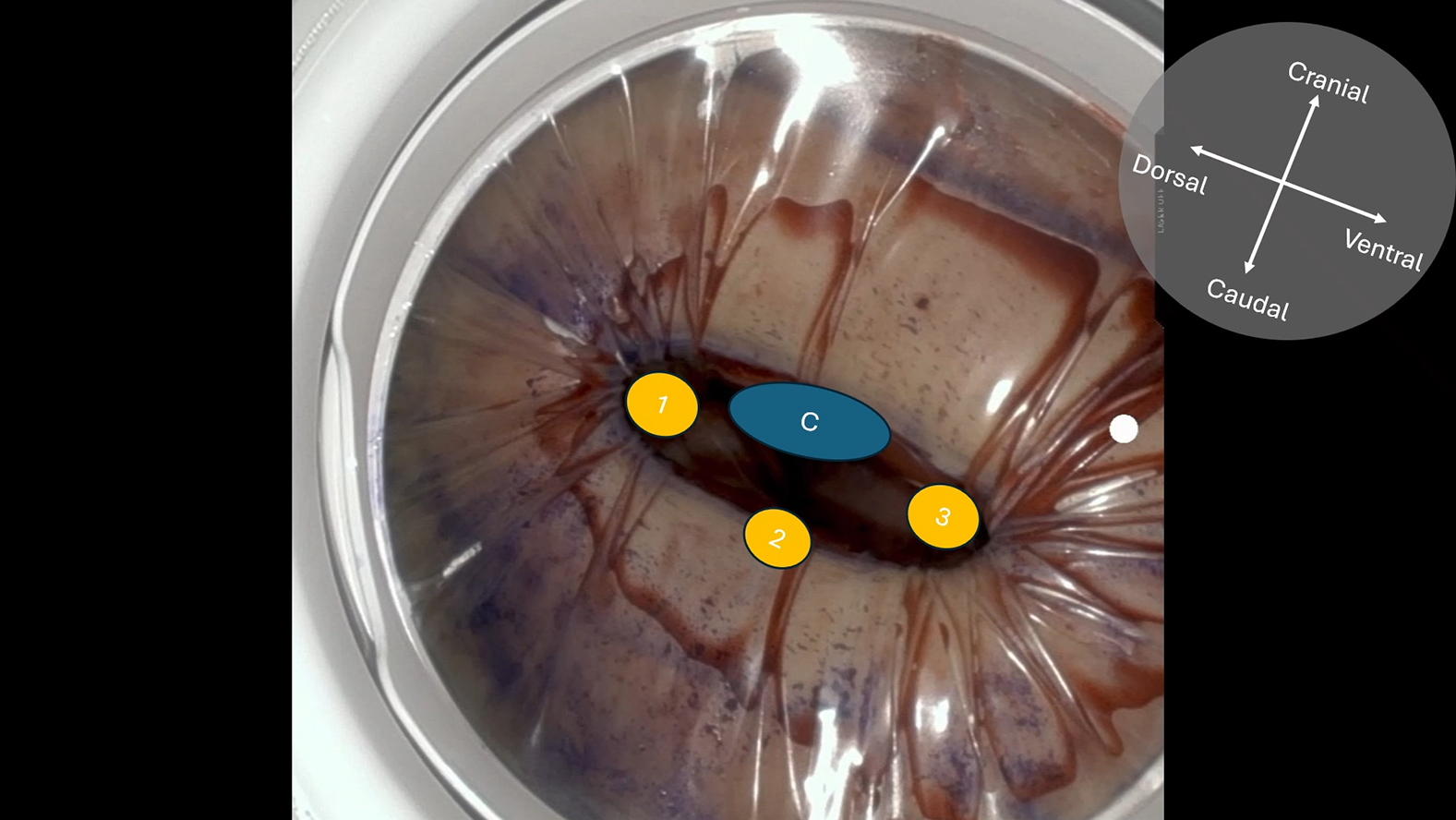

Supplement: Video 1 — Float docking technique. Video available at: https://www.jtcvs.org/article/S2666-2507(25)00549-8/fulltext. [file fx2.jpg]

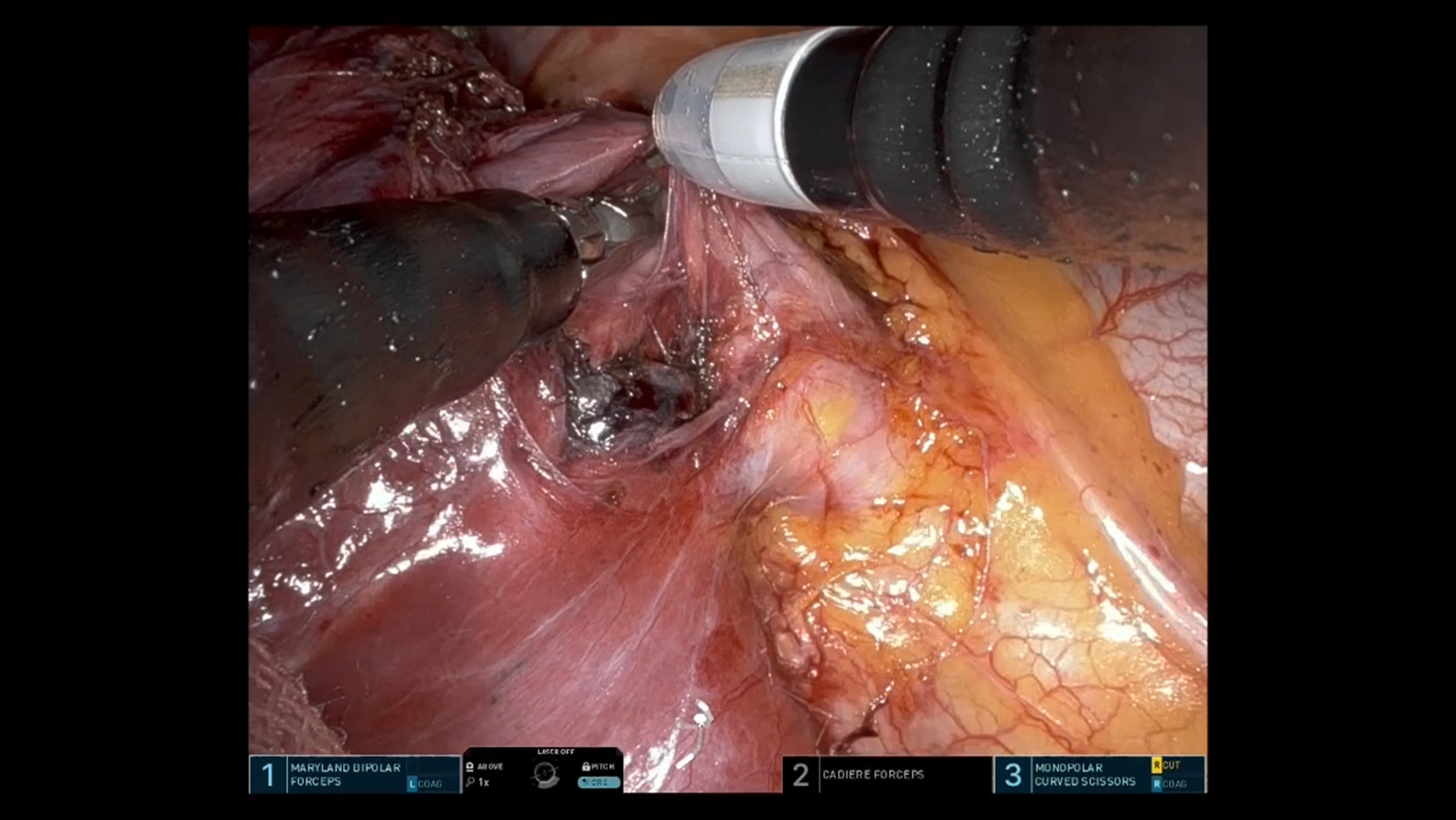

Supplement: Video 2 — Intercostal approach for single port robotic right upper lobectomy. Video available at: https://www.jtcvs.org/article/S2666-2507(25)00549-8/fulltext. [file fx3.jpg]
